# Case Report: Unilateral Sixth Cranial Nerve Palsy Associated With COVID-19 in a 2-year-old Child

**DOI:** 10.3389/fped.2021.756014

**Published:** 2021-12-17

**Authors:** Katrin Knoflach, Eva Holzapfel, Timo Roser, Lieselotte Rudolph, Marco Paolini, Maximilian Muenchhoff, Andreas Osterman, Matthias Griese, Matthias Kappler, Ulrich von Both

**Affiliations:** ^1^Department of Pediatrics, Dr. von Hauner Children's Hospital, University Hospital, Ludwig-Maximilians-University (LMU), Munich, Germany; ^2^Department of Ophthalmology, University Hospital Ludwig-Maximilians-University (LMU), Munich, Germany; ^3^Department of Radiology, University Hospital Ludwig-Maximilians-University (LMU), Munich, Germany; ^4^Max von Pettenkofer Institute & Gene Center, Virology, National Reference Center for Retroviruses, Ludwig-Maximilians-University (LMU), Munich, Germany; ^5^German Center for Infection Research, Partner Site Munich, Munich, Germany; ^6^German Center for Lung Research (DZL), Partner Site Munich, Munich, Germany

**Keywords:** COVID-19, SARS-CoV-2, sixth cranial nerve palsy, pediatric, anti-SARS-CoV-2 antibodies

## Abstract

Children have been described to show neurological symptoms in acute coronavirus disease 2019 (COVID-19) and multisystemic inflammatory syndrome in children (MIS-C). We present a 2-year-old boy's clinical course of unilateral acute sixth nerve palsy in the context of severe acute respiratory syndrome coronavirus 2 (SARS-CoV-2) infection. Onset of the palsy in the otherwise healthy boy occurred seven days after symptoms attributed to acute infection had subsided respectively 3 weeks after onset of respiratory symptoms. SARS-CoV-2 specific IgG was detected in serum as well as in cerebrospinal fluid. The patient showed a prolonged but self-limiting course with a full recovery after three and a half months. This case illustrates in a detailed chronological sequence that sixth cranial nerve involvement may occur as post-infectious, self-limiting complication of pediatric SARS-CoV-2-infection thus expanding the neurological spectrum of symptoms for children with COVID-19. Clinicians should be aware of the possibility of post-infectious sixth nerve palsy related to SARS-CoV-2-infection particularly in view of recent respiratory tract infection or confirmed cases of SARS-CoV-2-infection amongst the patient's close contacts.

## Introduction

Severe acute respiratory syndrome coronavirus 2 (SARS-CoV-2) infections, emerging first in the Chinese city of Wuhan in December 2019, may lead to coronavirus disease 2019 (COVID-19) (1). Generally mild or moderate respiratory disease is attributed to affected pediatric patients. The most common symptoms in children include fever, cough, shortness of breath, diarrhea, myalgia, headache and sore throat; severe pneumonia requiring oxygen support or critical illness as observed in the adult population is rare (1).

SARS-CoV-2 may also lead to neurological symptoms. Neurological symptoms have predominantly been reported in adults with up to 36% of patients (2). In the pediatric population, neurological symptoms may occur either as complication of acute infection with SARS-CoV-2 or in the context of associated multisystemic inflammatory syndrome in children (MIS-C) and include seizures, peripheral neuropathy, demyelination disorders, encephalopathy, Guillain-Barré syndrome and stroke. Some of these conditions may lead to altered mental status, weakness, fatigue and even long-term sequelae. In contrast to adult patients, anosmia and dysgeusia are rare (1, 3, 4).

Besides known involvement of the olfactory bulb, COVID-19 may also present with other cranial nerve dysfunctions including the optic, abducens, oculomotor and facial nerve as well as lower cranial nerves (5–7). There are only few reports of cranial nerve involvement for children (3, 6, 8–11). We present a 2-year-old boy with unilateral abducens nerve palsy as a likely post-infectious complication of COVID-19. Hereby, we want to show that cranial nerve involvement may occur as post-infectious complication of pediatric SARS-CoV-2-infection in addition to the previously described context of acute SARS-CoV-2-infection and MIS-C (3, 6, 8, 9, 12). In addition, this case lays special emphasis on pediatric sixth cranial nerve palsy in the context of SARS-CoV-2, a topic only discussed in one publication so far (3).

## Case Report

In January 2021, a 2-year-old boy of white Caucasian origin presented to his local ophthalmologist for acute unilateral sixth nerve palsy and was subsequently transferred to our pediatric emergency department for further evaluation. The patient, generally being fit and well, had developed a sudden dysfunction in lateral movement of his left eye, resulting in a continuous abduction deficit with consecutive fixated turn of the head to the left side. His medical history was unremarkable for trauma, headache, vomiting or fever. He had not received any vaccinations within the last few weeks. Apart from a mild gait instability, there were no concomitant symptoms or other focal neurological deficits on clinical examination. The patient did not suffer from any chronic diseases and did not take any regular medication; his vaccination status was complete according to national recommendations.

**Figure 1:**
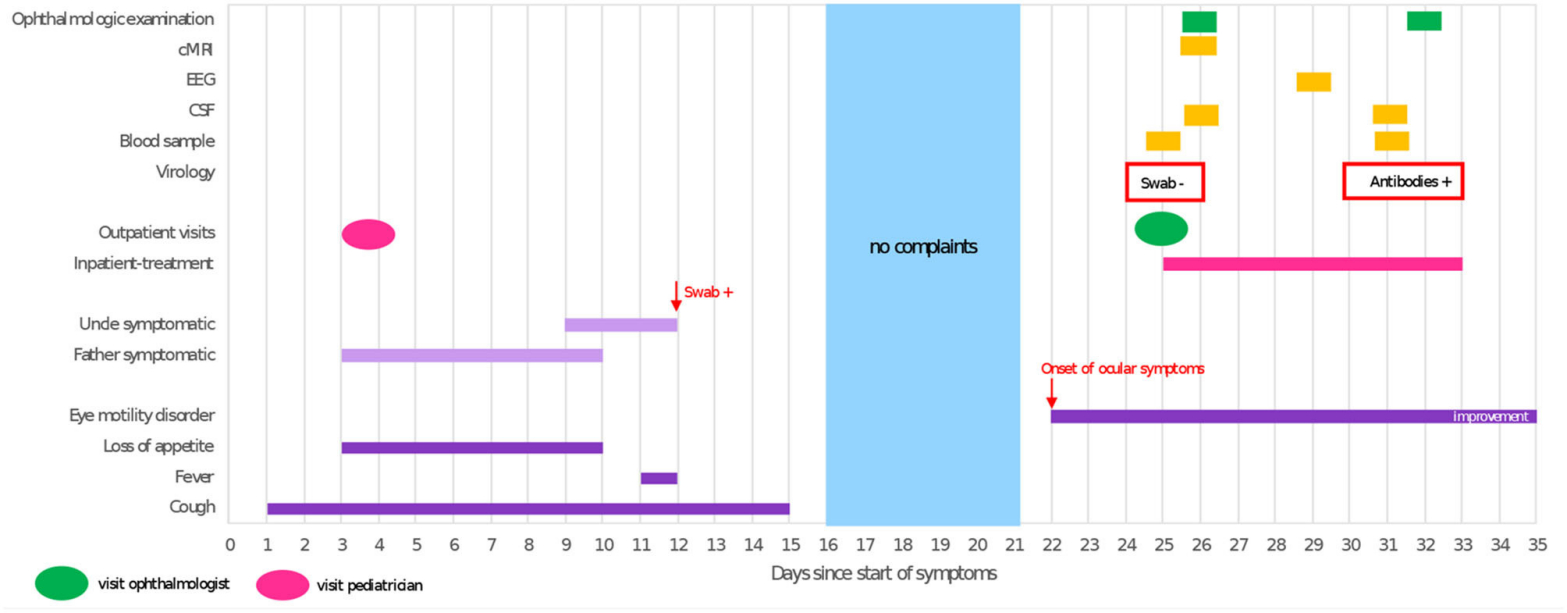
Timeline illustrating clinical course and diagnostic procedures. cMRI, cranial magnetic resonance imaging; EEG, electroencephalography; CSF, cerebrospinal fluid; swab, test result of oropharyngeal swab tested for SARS-CoV-2 RNA; +, positive; –, negative; antibodies, SARS-CoV-2 anti-spike IgG (Euroimmune, Germany).

Three weeks prior to onset of symptoms the patient had experienced a respiratory tract infection resulting in an increased respiratory rate, dry cough, intermittent fever and loss of appetite, lasting for 2 weeks. Symptomatic treatment was initiated by his local pediatrician, who attributed the patient's symptoms to a common cold rather than COVID-19. Thus, no oropharyngeal swab for SARS-CoV-2 or other viruses was obtained. At the same time, the patient's father and his uncle developed cough, dyspnea, sore throat and muscle aches; the uncle tested positive for SARS-CoV-2 on PCR from oropharyngeal swab (Figure 1). The child's uncle does not live in the same household but had been in close contact to the patient 4 days prior to his positive test for several hours due to an indoor-birthday party. The patient's relatives were unvaccinated as at that time the COVID-19 vaccines were still unavailable for the general public.

**Figure 2:**
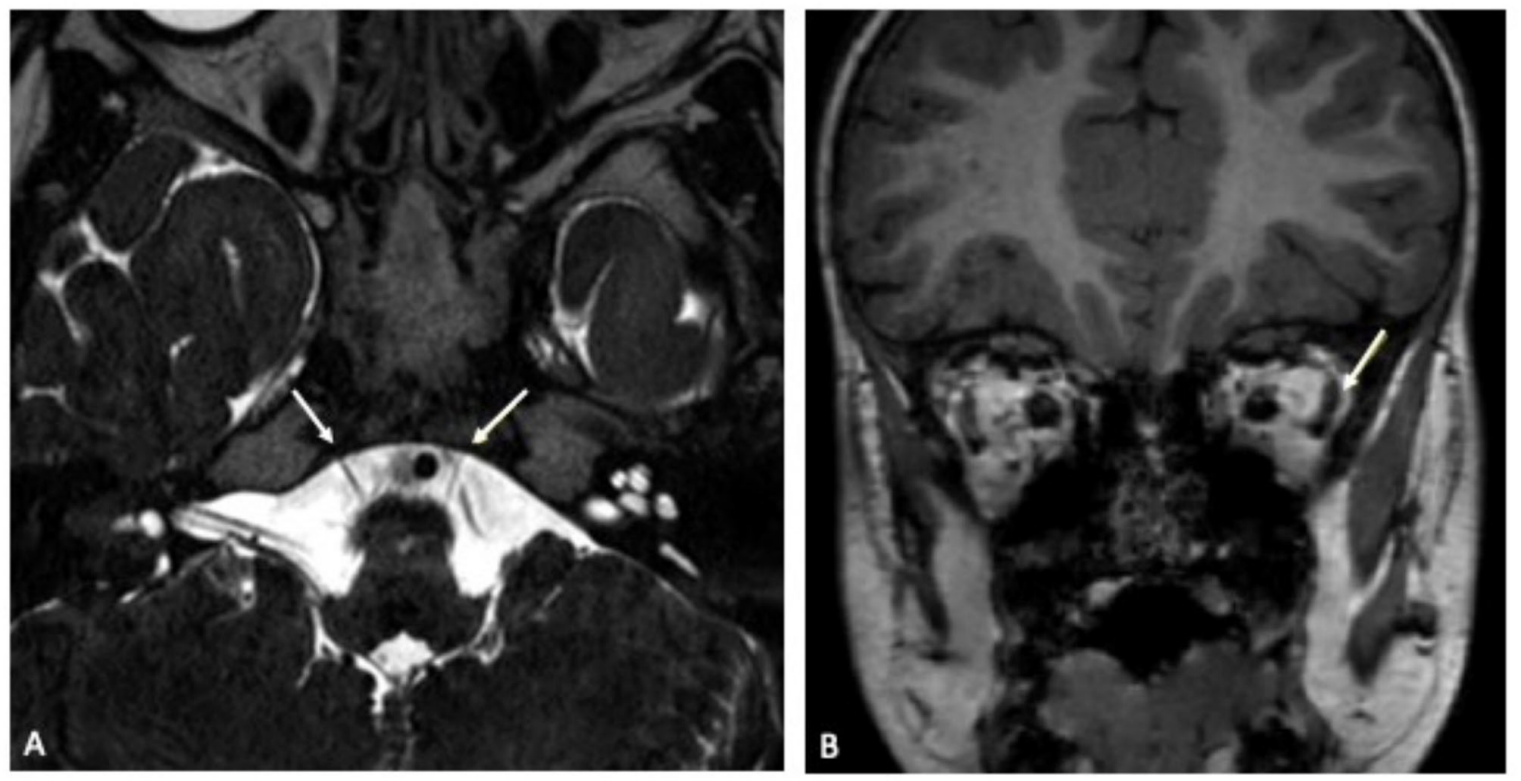
**(A)** Transverse plane of cranial MRI in constructive interference in steady state (CISS) sequence showing left-sided abducens nerve hypoplasia. Both abducens nerves are indicated by a white arrow. **(B)** Coronal plane of cranial MRI in native T1 sequence illustrating relative atrophy of the lateral rectus muscle (white arrow) in comparison to the contralateral right side.

On admission, laboratory inflammatory markers including C-reactive protein were negative. Full blood count showed mild thrombocytopenia (186 10^9/*l*^) but was unremarkable otherwise. Cranial contrast-enhanced magnetic resonance imaging (MRI) showed an hypoplastic left abducens nerve and atrophy of the corresponding left lateral rectus muscle compared to the contralateral side (Figure 2). There were no signs suggesting any inflammatory intracranial process or elevated intracranial pressure, no papilledema. A lumbar puncture was performed. The cerebrospinal fluid (CSF) opening pressure was 24 cmH_2_O corresponding to the upper limit of normal range (13) thus diagnostic lumbar puncture was followed by therapeutic drainage of 8 ml CSF. Routine CSF laboratory parameters yielded a normal result; no oligoclonal bands were detected on CSF/serum. Multiplex-PCR (Filmarray, BioFire, Biomerieux Lyon, France) from CSF was negative for cytomegalovirus (CMV), enterovirus, herpes simplex viruses 1 and 2, human herpesvirus 6, human parechovirus, varicella zoster virus, *Cryptococcus neoformans* and *gattii, E. coli* K1, *Haemophilus influenzae, Listeria monocytogenes, Neisseria meningitides* as well as *Streptococcus agalactiae* and *pneumoniae*. An additional multiplex-PCR performed on an oropharyngeal swab sample yielded a negative result for adenovirus, coronaviruses 229E, HKU1, NL63 and OC43, human metapneumovirus, human rhino-/ enterovirus, influenza virus A and B, Middle East Respiratory Syndrome Coronavirus (MERS-CoV), SARS-CoV-2, parainfluenza virus 1–4, respiratory syncytial virus, *Bordetella pertussis, Bordetella parapertusssis, Chlamydophila pneumonia* and *Mycoplasma pneumoniae*. Testing for *Borrelia burgdorferi* showed no antibodies in neither serum nor CSF. An EEG was unremarkable. Repeated ophthalmologic examinations revealed incomitant squint angles due to left-sided sixth nerve palsy and a significant abduction deficit of the left eye, consistent with the diagnosis of left abducens nerve palsy. An underlying retraction syndrome was considered unlikely due to the sudden onset of symptoms and absent globe retraction. Optic nerve examination was unremarkable.

Real-time reverse transcriptase PCR (rRT-PCR) test for SARS-CoV-2 (oropharyngeal swab sample) was negative on admission, while serology turned out to be positive for SARS-CoV-2 anti-spike IgG (Euroimmune, Germany). Of particular note in this context, SARS-CoV-2 specific IgG was also detected in CSF. Pathogen-specific antibody index as an indicator for potential intrathecal antibody production was negative, suggesting involvement of central nervous system being secondary to systemic infection rather than direct viral infection (14). An rRT-PCR for SARS-CoV-2 from CSF was negative.

Given the boy's history of recent respiratory tract infection, COVID-19 very likely in his father and proven in his uncle, and detection of SARS-CoV-2-IgG antibodies in the patient's serum and CSF, post-infectious abducens nerve palsy appeared to be the most likely diagnosis. During inpatient stay, symptoms already showed spontaneous mild improvement without therapeutic measures. Following discharge, the boy was regularly seen for ophthalmologic follow-ups. Three months following onset of abducens nerve palsy, the family noticed a distinct improvement in eye movement and the child eventually made a full recovery 2 weeks later.

## Discussion

Various pathologies may result in acute palsy of the abducens/sixth cranial nerve in children, such as trauma, inflammatory diseases (Guillain-Barré syndrome, multiple sclerosis), infections including meningitis, malignancy, sinus vein thrombosis or pseudotumor cerebri. Abducens nerve palsy clinically presents with diplopia, inward deviation of the eye and difficulty with lateral ocular movement. Accompanying symptoms such as headache, vomiting, unstable gait and fever may be present depending on the palsy's etiology (15).

Our patient had no history of trauma. Neurological examination was unremarkable besides unilateral abducens nerve palsy and did not reveal any other deficits or meningism, especially no ataxia or areflexia as seen in Miller-Fisher-syndrome. There were no hints for multiple sclerosis on neither CSF nor MRI; MRI did not show cerebrovascular disease/ thrombosis or an intracranial tumor. No viral or bacterial antigens could be detected using the Multiplex-PCR Filmarray CSF panel (BioFire, Biomerieux Lyon, France).

In the context of MIS-C, SARS-CoV-2 may lead to increased intracranial pressure in pediatric patients as described in two cases, both presenting with headache, diplopia and unilateral abducens nerve palsy resolving within days following lumbar puncture (16). Our patient showed a CSF opening pressure within the upper normal limits, no papilledema. As it seemed possible that intracranial pressure was elevated compared to the patient's usual condition, therapeutic drainage was performed. However, there was no remarkable improvement following the procedure, so pseudotumor cerebri seemed unlikely.

Signal alteration on MRI has already been described in patients presenting with cranial nerve involvement in COVID-19 (5). In our case, gadolinium enhancement was neither seen intracerebral nor in any portion of the abducens nerve. However, imaging revealed an hypoplastic abducens nerve on the affected side and ipsilateral atrophy of the innervated lateral rectus muscle suggestive of a potential virally mediated direct or indirect affection of the nerve. Due to these morphologic abnormalities, with horizontal eye movement dysfunction being a typical feature, underlying congenital Stilling-Turk-Duane syndrome/ Duane retraction syndrome was discussed (17). However, repeated ophthalmologic examinations showed no globe retraction with eyelid fissure narrowing, a frequent typical finding one would expect in Duane retraction syndrome due to a paradoxical anomaly in lateral rectus innervation (18). Because of alternate innervation, the lateral rectus muscle in Stilling-Turk-Duane syndrome would not show atrophy as was seen in our patient (19). Furthermore, the boy showed a sudden onset of symptoms with no past medical history of strabismus, difficulty in eye movement or ophthalmologic disease. Therefore, manifestation of an underlying congenital Stilling-Turk-Duane syndrome was considered to be very unlikely.

Interestingly, a similar morphologic picture was recently described in a 32-year-old male showing acute unilateral abducens nerve palsy related to COVID-19 (20). MRI revealed ipsilateral atrophy and hyperintensity of the lateral rectus muscle 5 weeks after onset of diplopia, representing a likely correlate for denervation of the abducens nerve due to a direct or indirect virus-mediated insult. In contrast to our patient and other case reports about SARS-CoV-2 associated cranial nerve palsies (5, 8, 9), this patient's palsy did not improve over time.

We hypothesize that our patient's abducens nerve palsy was a temporary, self-resolving para- or post-infectious complication of COVID-19. Pediatric cranial nerve palsy following viral or bacterial infection or even in the context of immunizations is a well-known phenomenon, especially for Bell's palsy (9, 21, 22). In children, cranial nerve involvement has been described related to acute COVID-19 or MIS-C (3, 8, 9) including one case with third cranial nerve palsy following MIS-C after an asymptomatic period (12). MIS-C was unlikely in our patient given the absence of systemic inflammatory signs (3). There is one pediatric case report about a child presenting with third nerve palsy in a presumed context of SARS-CoV-2-infection due to detection of SARS-CoV-2-IgM in patient's serum. However, no IgG antibodies were detected and infection was not confirmed by follow-up serology, so this case may not represent a COVID 19 associated pathology (6). For adults, few cases have already been described showing a delayed manifestation of cranial nerve dysfunction after SARS-CoV-2 infection similar to our case (23). While some children have been described to only show ophthalmoparesis as symptom of SARS-CoV-2-infection (6), adults tend to show additional (preceding) symptoms such as anosmia, respiratory or general complaints (23, 24). For the majority of both adult and pediatric cases, rapid recovery within 4 to 6 weeks has been reported (6, 20, 23).

Several mechanisms have been proposed to explain central nervous system (CNS) involvement in COVID-19 and MIS-C, such as direct infection of the nervous system, vasculitis of corresponding blood vessels or inflammatory response secondary to local and/or systemic infection including molecular mimicry (3, 25, 26). Earlier reports describing results from *post mortem* analyses on deceased SARS-CoV infected patients with neurologic symptoms revealing SARS-CoV in cerebrospinal fluid and autoptic brain tissue seem to suggest that neurologic symptoms could be attributed to viral involvement of the CNS (27). Childhood COVID-19 or MIS-C is characterized by a strong immune-mediated inflammatory response (3). Acute and delayed SARS-CoV-2-related CNS abnormalities have been demonstrated to occur in children with imaging patterns being primarily of postinfectious immune-mediated origin (4). Similarly, immune-mediated or autoimmune mechanisms seem to be more likely than direct virus-induced damage in our patient since only SARS-CoV-2 IgG but no viral antigens were detected in CSF. Thus, such an immune-mediated damage may have resulted in the above-mentioned morphologic abnormalities of the sixth nerve visible on the patient's MRI; no follow-up MRI was performed in view of clinical improvement. No anti-inflammatory treatment was initiated; clinical symptoms gradually subsided and were thus self-limiting. Experience from similar courses related to other (suspected) post-infectious cranial nerve palsies has demonstrated that immunomodulators such as glucocorticoids or immunoglobulins might be a pharmacological treatment option (28, 29).

We conclude that pediatric COVID-19 may lead to isolated temporary para- or post-infectious neurological complications. Although children tend to show less severe respiratory symptoms, they may experience prolonged complaints as in our case. To our knowledge, this is the first detailed case report about post-infectious sixth nerve palsy in the context of pediatric SARS-CoV-2 infection. So far, similar cases have only been mentioned in one other study (3). As data about cranial nerve involvement in pediatric COVID-19 is rare and reports are primarily based on adult cases, we would like to raise awareness for this condition in children and advocate for increased vigilance of clinicians worldwide.

## Data Availability Statement

The datasets presented in this article are not readily available because they contain patient clinical data. Requests to access the datasets should be directed to the corresponding author.

## Ethics Statement

Written informed consent was obtained from the individual(s), and minor(s)' legal guardian/next of kin, for the publication of any potentially identifiable images or data included in this article.

## Author Contributions

KK and UB obtained parent's informed consent, wrote the first draft of the manuscript, and were responsible for patient's clinical care. EH, LR, MG, and MK were responsible for patient's clinical care and contributed to the final version of the manuscript. MP was responsible for imaging analysis and contributed to the final version of the manuscript. MM and AO were responsible for laboratory analysis concerning SARS-CoV-2 and contributed to the final version of the manuscript. TR was involved in patient's clinical care and contributed to the final version of the manuscript. All authors contributed to the article and approved the submitted version.

## Conflict of Interest

The authors declare that the research was conducted in the absence of any commercial or financial relationships that could be construed as a potential conflict of interest.

## Publisher's Note

All claims expressed in this article are solely those of the authors and do not necessarily represent those of their affiliated organizations, or those of the publisher, the editors and the reviewers. Any product that may be evaluated in this article, or claim that may be made by its manufacturer, is not guaranteed or endorsed by the publisher.
